# Supplementary material for: Llama and Alpaca Management in Germany—Results of an Online Survey among Owners on Farm Structure, Health Problems and Self-Reflection
Source: Animals (Basel). 2021 Jan 6;11(1):102. doi: 10.3390/ani11010102 (PMC7825519; doi:10.3390/ani11010102)
Supplement: Supplementary file 1 [file animals-11-00102-s001.pdf]

Document S1: complete survey results

Part A: General information *[Teil A: Allgemeine Angaben]*

1. Which federal state is your farm located in? (single choice. n=255)
[In welchem Bundesland befindet sich Ihr Betrieb? (Einfachantwort. n=255)]

- ➢ North Rhine-Westphalia *[Nordrhein-Westfalen]*: 49 (19.2%)
- ➢ Lower Saxony *[Niedersachsen]*: 47 (18.4%)
- ➢ Bavaria *[Bayern]*: 45 (17.6%)
- ➢ Baden-Württemberg *[Baden-Württemberg]*: 21 (8.2%)
- ➢ Hesse *[Hessen]*: 16 (6.3%)
- ➢ Brandenburg *[Brandenburg]*: 14 (5.5%)
- ➢ Schleswig-Holstein *[Schleswig-Holstein]*: 14 (5.5%)
- ➢ Rhineland-Palatinate *[Rheinland-Pfalz]*: 11 (4.3%)
- ➢ Saxony *[Sachsen]*: 11 (4.3%)
- ➢ Thuringia *[Thüringen]*: 10 (3.9%)
- ➢ Saxony-Anhalt *[Sachsen-Anhalt]*: 7 (2.7%)
- ➢ Mecklenburg-Vorpommern *[Mecklenburg-Vorpommern]*: 6 (2.4%)
- ➢ Saarland *[Saarland]*: 4 (1.6%)
- ➢ Berlin *[Berlin]*: 0 (0%)
- ➢ Bremen *[Bremen]*: 0 (0%)
- ➢ Hamburg *[Hamburg]*: 0 (0%)

2. What kind of South American camelids do you keep? (multiple choice. n=255)
[Was für Neuweltkameliden halten Sie? (Mehrfachantwort. n=255)]

a. Alpacas *[Alpakas]*
b. Llamas *[Lamas]*
c. Alpaca and llama crossbreeds *[Kreuzungen aus Lamas und Alpakas]*

- ➢ Alpacas only *[Nur Alpakas]*: 175 (68.6%)
- ➢ Llamas and alpacas *[Lamas und Alpakas]*: 42 (16.5%)
- ➢ Llamas only *[Nur Lamas]*: 27 (10.6%)
- ➢ Llamas, alpacas and crossbreeds *[Lamas, Alpakas und Kreuzungen]*: 3 (1.2%)
- ➢ Llamas and crossbreeds *[Lamas und Kreuzungen]*: 3 (1.2%)
- ➢ Alpacas and crossbreeds *[Alpakas und Kreuzungen]*: 2 (0.8%)
- ➢ Crossbreeds only *[Nur Kreuzungen]*: 0 (0%)
- ➢ No information *[Keine Angabe]*: 3 (1.2%)

3. What type of alpaca do you keep? (If "yes" to question 2a. Multiple choice. n=222)
[Welchen Alpakatyp halten Sie? (Wenn „ja" bei Frage 2a. Mehrfachantwort. n=222)]

- ➢ Huacaya *[Huacaya]*: 219 (98.6%)
- ➢ Suri *[Suri]*: 37 (16.7%)
- ➢ Other *[Sonstiges]*: 2 (0.9%)
    - "Huacaya/Suri-hybrid"

4. How many alpacas of which age group and sex do you keep? (If "yes" to question 2a. n=220)
[Wie viele Alpakas welcher Altersklasse und welchen Geschlechts halten Sie? (Wenn „ja" bei Frage 2a. n=220)]

- ➢ Females <1 year *[Stuten <1 Jahr]*: 240 (7.8%)
- ➢ Females 1-3 years *[Stuten 1-3 Jahre]*: 467 (15.3%)

- Females 4-10 years *[Stuten 4-10 Jahre]*: 830 (27.1%)
- Females >10 year *[Stuten >10 Jahre]*: 181 (5.9%)
- Males <1 year *[Hengste <1 Jahr]*: 235 (7.7%)
- Males 1-3 years *[Hengste 1-3 Jahre]*: 273 (8.9%)
- Males 4-10 years *[Hengste 4-10 Jahre]*: 305 (10.0%)
- Males >10 years *[Hengste >10 Jahre]*: 75 (2.4%)
- Castrated males <1 year *[Wallache <1 Jahr]*: 17 (0.6%)
- Castrated males 1-3 years *[Wallache 1-3 Jahre]*: 132 (4.3%)
- Castrated males 4-10 years *[Wallache 4-10 Jahre]*: 268 (8.8%)
- Castrated males >10 years *[Wallache >10 Jahre]*: 39 (1.3%)

5. Which type of llama do you keep? (If "yes" to question 2b. Multiple choice. n=75)
[Welchen Lamatyp halten Sie? (Wenn „ja" bei Frage 2b. Mehrfachantwort. n=75)]

- Woolly llama *[Woolly Lama]*: 49 (65.3%)
- Classic llama *[Classic Lama]*: 48 (64.0%)
- Suri llama *[Suri Lama]*: 21 (28.0%)
- Other *[Sonstiges]*: 9 (12.0%)
  - "Medium"; "Silky"; "Guanako"

6. How many llamas of which age group and sex do you keep? (If "yes" to question 2b. n=75)
[Wie viele Lamas welcher Altersklasse und welchen Geschlechts halten Sie? (Wenn „ja" bei Frage 2b. n=75)]

- Females <1 year *[Stuten <1 Jahr]*: 33 (4.5%)
- Females 1-3 years *[Stuten 1-3 Jahre]*: 71 (9.6%)
- Females 4-10 years *[Stuten 4-10 Jahre]*: 192 (25.9%)
- Females >10 year *[Stuten >10 Jahre]*: 75 (10.1%)
- Males <1 year *[Hengste <1 Jahr]*: 19 (2.6%)
- Males 1-3 years *[Hengste 1-3 Jahre]*: 49 (6.6%)
- Males 4-10 years *[Hengste 4-10 Jahre]*: 65 (8.8%)
- Males >10 years *[Hengste >10 Jahre]*: 34 (4.6%)
- Castrated males <1 year *[Wallache <1 Jahr]*: 2 (0.3%)
- Castrated males 1-3 years *[Wallache 1-3 Jahre]*: 29 (3.9%)
- Castrated males 4-10 years *[Wallache 4-10 Jahre]*: 117 (15.8%)
- Castrated males >10 years *[Wallache >10 Jahre]*: 55 (7.4%)

7. Do you have other farm animals among your livestock? (Multiple choice. n=255)
[Haben Sie andere Nutztiere in Ihrem Bestand? (Mehrfachantwort. n=255)]

- No other farm animals *[keine anderen Nutztiere]*: 113 (44.3%)
- Poultry *[Geflügel]*: 99 (38.8%)
- Horses *[Pferde]*: 46 (18.0%)
- Sheep *[Schafe]*: 29 (11.4%)
- Goats *[Ziegen]*: 25 (9.8%)
- Cattle *[Rinder]*: 21 (8.2%)
- Pigs *[Schweine]*: 16 (6.3%)
- Other *[Sonstiges]*: 28 (11.0%)
  - "Donkeys" *[Esel]*; "Rabbits" *[Kaninchen]*; "Deer" *[Hirsche]*; "Ducks" *[Enten]*; "Moufflon" *[Muffelwild]*; "Geese" *[Gänse]*; "Bactrian camels" *[Trampeltiere]*; "Kangaroos" *[Kängurus]*; "Guinea pigs" *[Meerschweinchen]*; "Bees" *[Bienen]*; "Dogs" *[Hunde]*
- No information *[keine Antwort]*: 6 (2.4%)

8. Since when have you kept South American camelids? (Single choice. n=254)
[Seit wann halten Sie Neuweltkameliden? (Einfachantwort. n=254)]

- 1986: 1
- 1997: 1
- 1999: 1
- 2000: 1
- 2001: 1
- 2002: 3
- 2003: 2
- 2004: 2
- 2005: 9
- 2006: 7
- 2007: 7
- 2008: 6
- 2009: 12
- 2010: 12
- 2011: 24
- 2012: 10
- 2013: 15
- 2014: 19
- 2015: 22
- 2016: 23
- 2017: 20
- 2018: 29
- 2019: 27

9. Which purposes do you keep your South American camelids for? (Multiple choice. n=255)
[Bitte geben Sie die Nutzungsrichtung Ihrer Neuweltkameliden an. (Mehrfachantwort. n=255)]

- Hobby *[Hobby]*: 164 (64.3%)
- Wool *[Wolle]*: 142 (55.7%)
- Breeding *[Zucht]*: 132 (51.8%)
- Trekking *[Trekking]*: 127 (49.8%)
- Therapy *[Therapie]*: 66 (25.9%)
- Landscape conservation *[Landschaftspflege]*: 50 (19.6%)
- Meat *[Fleischgewinnung]*: 0 (0%)
- Herd protection for sheep *[Herdenschutz für Schafe]*: 0 (0%)
- Other *[Sonstiges]*: 10 (3.9%)
    - "Animal-based intervention" *[tiergestützte Intervention]*; "Seminars for managers" *[Seminare für Führungskräfte]*; "Nature hikes" *[Naturwanderungen]*; "Children's birthday parties" *[Kindergeburtstage]*; "Tourism" *[Tourismus]*; "Paedagogy" *[Pädagogik]*; "Herd protection for chickens" *[Herdenschutz für Hühner]*

10. Are the camelids your main source of income? (Single choice. n=255)
[Dient die Neuweltkamelidenhaltung dem Haupterwerb? (Einfachantwort. n=255)]

- Yes *[Ja]*: 21 (8.2%)
- No, only on a sideline basis *[Nein, nur Nebenerwerb]*: 127 (49.8%)
- No income *[Nein, keine Einnahmen]*: 107 (42.0%)

11. How many people are involved in taking care of your camelids? (Single choice. n=255)
[Wie viele Personen sind in die Betreuung der Neuweltkameliden eingebunden? (Einfachantwort. n=255)]

- 1: 34 (13.3%)
- 2: 145 (56.9%)

- 3: 47 (18.4%)
- 4: 20 (7.8%)
- 5: 6 (2.4%)
- More than 5 *[Mehr als 5]*: 3 (1.2%)

Part B: Husbandry *[Teil B: Haltung]*

12. How do you receive information about the camelid husbandry? (Multiple choice. n=252)
[Wie informieren Sie sich über die Haltung von Neuweltkameliden? (Mehrfachantwort. n=252)]

- Books *[Bücher]*: 216 (85.7%)
- Seminars for further education *[Fortbildungen]*: 196 (77.8%)
- Llama/alpaca breeder *[ZüchterIn]*: 175 (69.4%)
- Web research *[Internetrecherche]*: 156 (61.9%)
- Veterinarian *[Tierarzt/Tierärztin]*: 144 (57.1%)
- Web forum *[Internetforen]*: 138 (54.8%)
- Journals *[Zeitschriften]*: 116 (46.0%)
- Family/friends *[Familie/Freunde]*: 45 (17.9%)
- Other *[Sonstiges]*: 19 (7.5%)
    - "Associations for South American camelids" *[Neuweltkamelvereine]*; "Shearer" *[Scherer]*; "Llama and alpaca shows" *[Lama-/Alpaka-Shows]*; "University of Veterinary Medicine Hannover, Foundation, Hannover, Germany" *[Stiftung Tierärztliche Hochschule Hannover]*; "Internships" *[Praktika]*; "Previous owner" *[Vorbesitzer]*; "Teaching material on horses" *[Lehrmaterial für Pferde]*

13. Do you keep your camelids in several groups? (Single choice. n=255)
[Halten Sie Ihre Neuweltkameliden in mehreren Gruppen? (Einfachantwort. n=255)]

- Yes *[Ja]*: 124 (48.6%)
- No *[Nein]*: 121 (47.5%)
- No information *[Keine Antwort]*: 10 (3.9%)

14. What is the average number of animals per group? (excluding small separate groups, e.g. small groups of males) (If "yes" to question 13. Single choice. n=124)
[Wie viele Tiere befinden sich durchschnittlich in einer Gruppe? (kleine separate Gruppen ausgenommen, z.B. Hengstgruppen) (Wenn „Ja" bei Frage 13. Einfachantwort. n=124)]

- <10 animals *[<10 Tiere]*: 72 (58.1%)
- 10-20 animals *[10-20 Tiere]*: 43 (34.7%)
- >20 animals *[>20 Tiere]*: 9 (7.3%)

15. Are your camelids kept together with another animal species/ other animal species? (Single choice. n=255)
[Werden Ihre Neuweltkameliden mit einer anderen Tierart / mit anderen Tierarten zusammen gehalten? (Einfachantwort. n=255)]

- Yes *[Ja]*: 17 (6.7%)
- No *[Nein]*: 235 (92.2%)
- No information *[Keine Antwort]*: 3 (1.2%)

16. Which animal species do you keep your camelids with? (If "yes" to question 15. Multiple choice. n=17)
[Mit welcher Tierart / mit welchen Tierarten halten Sie Ihre Neuweltkameliden zusammen? (Wenn „Ja" bei Frage 15. Mehrfachantwort. n=17)]

- Poultry *[Geflügel]*: 11 (64.7%)
- Sheep *[Schafe]*: 5 (29.4%)

- ➢ Goats *[Ziegen]*: 5 (29.4%)
- ➢ Cattle *[Rinder]*: 2 (11.8%)
- ➢ Pigs *[Schweine]*: 1 (5.9%)
- ➢ Horses *[Pferde]*: 1 (5.9%)
- ➢ Other *[Sonstiges]*: 2 (11.8%)
  - "Donkeys" *[Esel]*; "Dogs/cats" *[Hunde/Katzen]*

17. How do you house your camelids? (Single choice. n=255)
[Wie ist die Unterbringung Ihrer Neuweltkameliden? (Einfachantwort. n=255)]

- ➢ Stable and pasture *[Stall und Weide]*: 140 (54.9%)
- ➢ Pasture/paddock with shelter *[Weide / Paddock mit Unterstand]*: 93 (36.5%)
- ➢ Stable with paddock *[Stall mit Außenbereich]*: 18 (7.1%)
- ➢ Pasture *[Weide]*: 1 (0.4%)
- ➢ Stable *[Stall]*: 1 (0.4%)
- ➢ No information *[keine Antwort]*: 2 (0.8%)

18. What´s the approximate size of the stable? (If "stable", "stable with paddock", "stable and pasture" at question 17. Single choice. n=152)
[Wie groß ist der Stall ungefähr? (Wenn „Stall", „Stall mit Außenbereich", „Stall und Weide" bei Frage 17. Einfachantwort. n=152)]

- ➢ Minimum: 5m^2
- ➢ Maximum: 500m^2
- ➢ Mean value *[Mittelwert]*: 66.3m^2
- ➢ Median: 43m^2

19. What sort of flooring/litter do you have in the stables? (If "stable", "stable with paddock", "stable and pasture" at question 17. Multiple choice. n=159)
[Welchen Fußboden / welche Einstreu haben Sie in den Stallungen? (Wenn „Stall", „Stall mit Außenbereich", „Stall und Weide" bei Frage 17. Mehrfachantwort. n=159)]

- ➢ Concrete *[Beton]*: 104 (65.4%)
- ➢ Straw *[Stroh]*: 86 (54.1%)
- ➢ Rubber mats *[Gummimatten]*: 51 (32.1%)
- ➢ Wood shavings *[Sägespäne]*: 30 (18.9%)
- ➢ Hay *[Heu]*: 24 (15.1%)
- ➢ Soil *[Erde]*: 23 (14.5%)
- ➢ Wooden floor *[Holz]*: 12 (7.5%)
- ➢ Sand *[Sand]*: 10 (6.3%)
- ➢ Other *[Sonstiges]*: 22 (13.8%)
  - "(Wood-, straw-, miscanthus-) pellets" *[Holz-, Stroh-, Miscanthus-Pellets]*; "Straw meal" *[Strohmehl]*; "Hemp bedding" *[Hanfstreu]*; "Paving stones" *[Pflastersteine]*; "Flax bedding" *[Leinenstroh]*; "Chopped straw" *[Strohhäcksel]*; "Concrete slabs" *[Betonplatten]*; "Gravel" *[Schotter]*; "Brick" *[Klinker]*; "Stone" *[Steine]*

20. How large is the pasture / the paddock? If there are several pastures, indicate the average size of one pasture. (If "pasture", "pasture/paddock with shelter", "stable with paddock", "stable and pasture" at question 17. Single choice. n=244)
[Wie groß ist Ihre Weide / Auslauffläche ungefähr? Wenn mehrere Weiden vorhanden, dann Angabe der durchschnittlichen Größe einer Weide. (Wenn „Weide", „Weide / Paddock mit Unterstand", „Stall mit Außenbereich", „Stall und Weide" bei Frage 17. Einfachantwort. n=244)]

- ➢ Minimum: 4m^2

- Maximum: 110,000m^2
- Mean value *[Mittelwert]*: 8961.2m^2
- Median: 5000m^2

21. How many pastures/paddocks do you have for your camelids? (If "pasture", "pasture/paddock with shelter", "stable with paddock", "stable and pasture" at question 17. Single choice. n=252)
[Wie viele verschiedene Weiden / Auslaufflächen stehen den Neuweltkameliden zur Verfügung? (Wenn „Weide", „Weide / Paddock mit Unterstand", „Stall mit Außenbereich", „Stall und Weide" bei Frage 17. Einfachantwort. n=252)]

- 1: 14 (5.6%)
- 2: 63 (25.0%)
- 3: 71 (28.2%)
- 4: 44 (17.5%)
- 5: 19 (7.5%)
- 6: 12 (4.8%)
- 7: 13 (5.2%)
- 8: 8 (3.2%)
- 9: 1 (0.4%)
- 10: 4 (1.6%)
- More than 10 *[Mehr als 10]*: 2 (0.8%)
- No information *[Keine Antwort]*: 1 (0.4%)

22. Do the camelids change pastures (rotation system)? (If "pasture", "pasture/paddock with shelter", "stable with paddock", "stable and pasture" at question 17. Single choice. n=252)
[Wechseln die Neuweltkameliden die Weide-/Auslaufflächen (Rotationssystem)? (Wenn „Weide", „Weide / Paddock mit Unterstand", „Stall mit Außenbereich", „Stall und Weide" bei Frage 17. Einfachantwort. n=252)]

- Yes *[Ja]*: 198 (78.6%)
- No *[Nein]*: 44 (17.5%)
- No information *[keine Antwort]*: 10 (4.0%)

Part C: Feeding *[Teil C: Fütterung]*

23. What is the main feed in summer? (Multiple choice. n=253)
[Welches Grundfutter / Hauptfutter füttern Sie im Sommer? (Mehrfachantwort. n=253)]

- Pasture grass *[Weidegras]*: 248 (98.0%)
- Hay *[Heu]*: 238 (94.1%)
- Straw *[Stroh]*: 15 (5.9%)
- Haylage *[Heulage]*: 3 (1.2%)
- Silage *[Grassilage]*: 1 (0.4%)
- Hay made from reed *[Reetheu]*: 1 (0.4%)
- Other *[Sonstiges]*: 2 (0.8%)
    - "Grass pellets" *[Graspellets]*; "Lucerne" *[Luzerne]*

24. What is the main feed in winter? (Multiple choice. n=253)
[Welches Grundfutter / Hauptfutter füttern Sie im Winter? (Mehrfachantwort. n=253)]

- Hay *[Heu]*: 250 (98.8%)
- Pasture grass *[Weidegras]*: 108 (42.7%)
- Straw *[Stroh]*: 22 (8.7%)
- Haylage *[Heulage]*: 7 (2.8%)
- Silage *[Grassilage]*: 2 (0.8%)
- Hay made from reed *[Reetheu]*: 1 (0.4%)

- Other *[Sonstiges]*: 23 (9.1%)
    - "Lucerne (-hay; -pellets)" *[Luzerne (-Heu; -Pellets)]*; "Willow branches" *[Weidenäste]*; "Aftergrass" *[Öhmd]*; "Grass pellets" *[Graspellets]*; "Pressed beet pulp" *[gepresste Rübenschnitzel]*; "Dried herbs" *[getrocknete Kräuter]*

25. How much basic feed do you feed the animals (main feed)? (Single choice. n=255)
[Wie viel Grundfutter füttern Sie? (Einfachantwort. n=255)]

- Ad libitum *[zur freien Verfügung]*: 233 (91.4%)
- A limited amount of feed *[begrenzte Futtermenge]*: 19 (7.5%)
- No information *[keine Antwort]*: 3 (1.2%)

26. Do you add other components to the main diet? (Multiple choice. n=251)
[Füttern Sie andere Futtermittel zusätzlich zu? (Mehrfachantwort. n=251)]

- No additional feed *[es wird nichts weiter zugefüttert]*: 8 (3.2%)
- Mineral feed *[Mineralfutter]*: 216 (86.1%)
- Concentrate *[Kraftfutter]*: 107 (42.6%)
- Vegetables *[Gemüse]*: 23 (9.2%)
- Fruit *[Obst]*: 14 (5.6%)
- Other *[Sonstiges]*: 47 (18.7%)
    - "Lucerne" *[Luzerne]*; "Herbs" *[Kräuter]*; "Corn cobs" *[Maiscobs]*; "Beet pulp" *[Rübenschnitzel]*; "Treacle" *[Melasse]*; "Linseed cake" *[Leinkuchen]*; "Wheat bran" *[Weizenkleie]*; "Leaves" *[Blätter]*

27. Does the concentrate or mineral feed contain vitamin D? (If "concentrate", "mineral feed" at question 26. Single choice. n=235)
[Ist dem Kraftfutter oder Mineralfutter Vitamin D zugesetzt? (Wenn „Kraftfutter", „Mineralfutter" bei Frage 26. Einfachantwort. n=235)]

- Yes *[Ja]*: 151 (64.3%)
- No *[No]*: 40 (17.0%)
- No information *[keine Antwort]*: 44 (18.7%)

28. If known: How much vitamin D is contained in the concentrate/mineral feed per kg dry matter (indicating the unit)? (If "yes" at question 27. Free text. n=151)
[Falls bekannt: Wie viel Vitamin D ist in dem Kraft- bzw. Mineralfutter pro kg Trockensubstanz (TS) enthalten (mit Angabe der Einheit)? (Wenn „Ja" bei Frage 27. Freitext. n=151)]

- Answered *[Anwort gegeben]*: 41 (27.2%)
    - Minimum: 1,400 IU per kg dry matter
    - Maximum: 108,000 IU per kg dry matter
- No information *[keine Antwort]*: 110 (72.8%)

Part D: Management *[Teil D: Management]*

29. Are new animals regularly added to the stock (own offspring excluded)? (Single choice. n=255)
[Kommen regelmäßig neue Tiere in den Bestand? (Eigene Nachzucht ausgenommen) (Einfachantwort. n=255)]

- Yes *[Ja]*: 37 (14.5%)
- No *[Nein]*: 211 (82.7%)
- No information *[keine Antwort]*: 7 (2.7%)

30. Where do new animals come from? (If "yes" to question 29. Multiple choice. n=37)
[Woher kommen neue Tiere? (Wenn „Ja" bei Frage 29. Mehrfachantwort. n=37)]

- ➢ Different stocks in Germany *[von verschiedenen Betrieben in Deutschland]*: 30 (81.1%)
- ➢ Stocks from abroad (EU) *[von Betrieben aus dem EU-Ausland]*: 9 (24.3%)
- ➢ Stocks from abroad (outside the EU) *[von Betrieben aus dem Nicht-EU-Ausland]*: 9 (24.3%)
- ➢ One other stock in Germany *[von einem einzigen anderen Betrieb in Deutschland]*: 2 (5.4%)

31. Are new animals kept separately for some time before joining the flock? (If "yes" at question 29. Single choice. n=37)
[Werden neue Tiere vor Eingliederung in die Herde für einige Zeit separat gehalten? (Wenn „Ja" bei Frage 29. Einfachantwort. n=37)]

- ➢ No *[Nein]*: 19 (51.4%)
- ➢ Yes *[Ja]*: 18 (48.6%)

32. How long are the new animals kept separately? (If "yes" at question 31. Single choice. n=18)
[Wie lange werden die Tiere separat gehalten? (Wenn „Ja" bei Frage 31. Einfachantwort. n=18)]

- ➢ <1 week *[<1 Woche]*: 2 (11.1%)
- ➢ 1 week *[1 Woche]*: 0 (0%)
- ➢ 2 weeks *[2 Wochen]*: 8 (44.4%)
- ➢ 3 weeks *[3 Wochen]*: 2 (11.1%)
- ➢ 4 weeks *[4 Wochen]*: 3 (16.7%)
- ➢ 5 weeks *[5 Wochen]*: 0 (0%)
- ➢ 6 weeks *[6 Wochen]*: 1 (5.6%)
- ➢ >6 weeks *[>6 Wochen]*: 2 (11.1%)

33. Do you have proof of origin for your camelids (pedigree)? (Single choice. n=255)
[Haben Sie einen Verwandtschaftsstatus / Herkunftsnachweise Ihrer Neuweltkameliden (Ahnentafel)? (Einfachantwort. n=255)]

- ➢ Yes *[Ja]*: 176 (69.0%)
- ➢ For certain animals only *[nur von bestimmten Tieren]*: 44 (17.3%)
- ➢ No *[Nein]*: 30 (11.8%)
- ➢ No information *[keine Antwort]*: 5 (2.0%)

34. Do you mark your animals (animal identification) and if so, how? (Multiple choice. n=245)
[Kennzeichnen Sie Ihre Tiere (Tieridentifikation) und wenn ja, wie? (Mehrfachantwort. n=245)]

- ➢ No marking *[keine Kennzeichnung]*: 45 (18.4%)
- ➢ Transponder (chip): 199 (81.2%)
- ➢ Ear tag *[Ohrmarke]*: 4 (1.6%)
- ➢ Other *[Sonstiges]*: 4 (1.6%)
  - "DNA-profile" *[DNA-Profil]*

35. How often do you shear the alpacas? (If "a" at question 2. Single choice. n=222)
[Wie oft scheren Sie Ihre Alpakas? (Wenn „a" bei Frage 2. Einfachantwort. n=222)]

- ➢ Never *[nie]*: 0 (0%)
- ➢ Every other year *[alle 2 Jahre]*: 2 (0.9%)
- ➢ Once a year *[1x pro Jahr]*: 217 (97.7%)
- ➢ Twice a year *[2x pro Jahr]*: 0 (0%)
- ➢ More than twice a year *[mehr als 2x pro Jahr]*: 0 (0%)
- ➢ Other *[Sonstiges]*: 1 (0.5%)

- "Huacaya-alpacas once a year, Suri-alpacas every 1-2 years" *[Huacaya-Alpakas 1x jährlich, Suri-Alpakas alle 1-2 Jahre]*

➢ No information *[keine Antwort]*: 2 (0.9%)

36. How often do you shear the llamas? (If "b" at question 2. Single choice. n=75)
[Wie oft scheren Sie Ihre Lamas? (Wenn „b" bei Frage 2. Einfachantwort. n=75)]

➢ Never *[nie]*: 0 (0%)
➢ Every other year *[alle 2 Jahre]*: 8 (10.7%)
➢ Once a year *[1x pro Jahr]*: 59 (78.7%)
➢ Twice a year *[2x pro Jahr]*: 0 (0%)
➢ More than twice a year *[mehr als 2x pro Jahr]*: 0 (0%)
➢ Other *[Sonstiges]*: 6 (8.0%)
- "Variable time periods" *[variable Zeitspannen]*

➢ No information *[keine Antwort]*: 2 (2.7%)

37. Who shears your animals? (If "more than twice a year", "twice a year", "once a year", every other year", "other" at question 35/36. Multiple choice. n=249)
[Wer führt die Schur durch? (Wenn „Mehr als 2x pro Jahr", „2x pro Jahr", „1x pro Jahr", „alle 2 Jahre", „Sonstiges" bei Frage 35/36. Mehrfachantwort. n=249)]

➢ Mobile shearing service *[mobile Scherservice]*: 152 (61.0%)
➢ Is performed by yourself *[wird selbst durchgeführt]*: 102 (41.0%)
➢ Other *[Sonstiges]*: 16 (6.4%)
- "Llama-/alpaca-breeder" *[Züchter]*; "Veterinarian" *[Tierarzt]*

38. How are your animals fixed during shearing? (If "more than twice a year", "twice a year", "once a year", every other year", "other" at question 35/36. Multiple choice. n=249)
[Wie werden Ihre Tiere während der Schur fixiert? (Wenn „Mehr als 2x pro Jahr", „2x pro Jahr", „1x pro Jahr", „alle 2 Jahre", „Sonstiges" bei Frage 35/36. Mehrfachantwort. n=249)]

➢ Fixation on the shearing table *[Fixation auf dem Schertisch]*: 112 (45.0%)
➢ Fixation on the ground *[Fixation auf dem Boden]*: 88 (35.3%)
➢ Fixation in standing position *[Fixation im Stehen]*: 52 (20.9%)
➢ Animals are tied up *[Tiere werden ausgebunden]*: 12 (4.8%)
➢ Other *[Sonstiges]*: 31 (12.4%)
- "Without fixation" *[ohne Fixation]*; "Loosely tied" *[locker angebunden]*

➢ No information *[keine Antwort]*: 2 (0.8%)

39. Do you cut your animals' toenails? (Single choice. n=255)
[Werden die Zehennägel der Tiere manchmal geschnitten? (Einfachantwort. n=255)]

➢ Yes *[Ja]*: 242 (94.9%)
➢ No *[Nein]*: 6 (2.4%)
➢ No information *[keine Antwort]*: 7 (2.7%)

40. Who cuts the toenails of your animals? (If "yes" to question 39. Multiple choice. n=242)
[Wer schneidet die Zehennägel Ihrer Neuweltkameliden? (Wenn „Ja" bei Frage 39. Mehrfachantwort. n=242)]

➢ Performed by yourself *[wird selbst durchgeführt]*: 222 (91.7%)
➢ Veterinarian *[Tierarzt/Tierärztin]*: 13 (5.4%)
➢ Llama/alpaca breeder *[ZüchterIn]*: 12 (5.0%)
➢ Shearer *[SchererIn]*: 11 (4.5%)
➢ Other *[Sonstiges]*: 3 (1.2%)

- “Claw trimmer” *[Klauenpfleger]*; “Friends/relatives” *[Freunde/Bekannte]*

41. How often do you cut the toenails of your camelids? (If “yes” to question 39. Single choice. n=242)
[Wie oft schneiden Sie die Nägel? (Wenn „Ja“ bei Frage 39. Einfachantwort. n=242)]

➢ If needed *[bei Bedarf]*: 100 (41.3%)
➢ More than twice a year *[mehr als 2x pro Jahr]*: 92 (38.0%)
➢ Twice a year *[2x pro Jahr]*: 18 (7.4%)
➢ Once a year *[1x pro Jahr]*: 13 (5.4%)
➢ Every other year *[alle 2 Jahre]*: 0 (0%)
➢ Other *[Sonstiges]*: 19 (7.9%)
  - “Variable time periods” *[variable Zeitspannen]*; “Light-coloured animals more often than dark-coloured animals” *[helle Tiere häufiger als dunkle Tiere]*

42. Do you sometimes shorten the fighting teeth of your adult males? (Single choice. n=255)
[Werden in Ihrem Bestand manchmal die Kampfzähne bei adulten Hengsten gekürzt? (Einfachantwort. n=255)]

➢ Yes *[Ja]*: 120 (47.1%)
➢ No *[Nein]*: 120 (47.1%)
➢ No information *[keine Antwort]*: 15 (5.9%)

43. Who performs this surgery? (If “yes” to question 42. Multiple choice. n=120)
[Wer führt diesen Eingriff durch? (Wenn „Ja“ bei Frage 42. Mehrfachantwort. n=120)]

➢ Veterinarian *[Tierarzt/Tierärztin]*: 67 (55.8%)
➢ Performed by yourself *[wird selbst durchgeführt]*: 25 (20.8%)
➢ Shearer *[SchererIn]*: 24 (20.0%)
➢ Llama/alpaca breeder *[ZüchterIn]*: 10 (8.3%)
➢ Other *[Sonstiges]*: 2 (1.7%)
  - “Dental technician” *[Zahntechniker]*; “Animal keeper” *[Tierpfleger]*
➢ No information *[keine Antwort]*: 4 (3.3%)

44. Do you vaccinate your camelids? (Single choice. n=255)
[Werden Ihre Tiere geimpft? (Einfachantwort. n=255)]

➢ Yes *[Ja]*: 215 (84.3%)
➢ No *[Nein]*: 31 (12.2%)
➢ No information *[keine Antwort]*: 9 (3.5%)

45. What are they vaccinated against? (If “yes” at question 44. Multiple choice. n=215)
[Wogegen werden Ihre Neuweltkameliden geimpft? (Wenn „Ja“ bei Frage 44. Mehrfachantwort. n=215)]

➢ Clostridial infections in general *[Clostridieninfektionen allgemein]*: 169 (78.6%)
➢ Not known *[unklar]*: 22 (10.2%)
➢ Tetanus in particular *[speziell Tetanus]*: 17 (7.9%)
➢ Chlamydial abortion *[Chlamydienabort]*: 17 (7.9%)
➢ Bluetongue disease *[Blauzungenkrankheit]*: 16 (7.4%)
➢ Rabies *[Tollwut]*: 11 (5.1%)
➢ Other *[Sonstiges]*: 0 (0%)
➢ No information *[keine Antwort]*: 8 (3.7%)

46. How often do you vaccinate your camelids? (If “yes” at question 44. Multiple choice, comments optional. n=215)
[Wie häufig impfen Sie? (Wenn „Ja“ bei Frage 44. Mehrfachantwort mit Kommentarfunktion. n=215)]

- Three times a year *[3x jährlich]*: 2 (0.9%)
- Twice a year *[2x jährlich]*: 14 (6.5%)
- Once a year *[1x jährlich]*: 184 (85.6%)
- Every other year *[alle 2 Jahre]*: 11 (5.1%)
- Once in a lifetime *[1x im Leben]*: 3 (1.4%)
- Other *[Sonstiges]*: 5 (2.3%)
    - "Decision of the vet" *[nach Ansage des Tierarztes]*; "Every five years" *[alle 5 Jahre]*
- No information *[keine Antwort]*: 5 (2.3%)

47. Do you deworm your camelids? (Single choice. n=255)
[Werden Ihre Tiere entwurmt? (Einfachantwort. n=255)]

- Yes *[Ja]*: 228 (89.4%)
- No *[Nein]*: 13 (5.1%)
- No information *[keine Antwort]*: 14 (5.5%)

48. How do you deworm your camelids? (If "yes" at question 47. Multiple choice. n=228)
[Wie entwurmen Sie Ihre Tiere? (Wenn „Ja" bei Frage 47. Mehrfachantwort. n=228)]

- By injection *[per Injektion]*: 152 (66.7%)
- Oral application *[orale Eingabe]*: 119 (52.2%)
- Other *[Sonstiges]*: 10 (4.4%)
    - "Alternately oral application and injection" *[oral und per Injektion im Wechsel]*; "For application on the skin" *[zum Auftragen auf die Haut]*; "Worm herbs" *[Wurmkräuter]*
- No information *[keine Angabe]*: 6 (2.6%)

49. How frequently do you deworm them? (If "yes" at question 47. Single choice. n=228)
[Wie häufig entwurmen Sie? (Wenn „Ja" bei Frage 47. Einfachantwort. n=228)]

- Less than once a year *[seltener als 1x pro Jahr]*: 3 (1.3%)
- Once a year *[1x pro Jahr]*: 26 (11.4%)
- Twice a year *[2x pro Jahr]*: 42 (18.4%)
- Three times a year *[3x pro Jahr]*: 13 (5.7%)
- Four times a year *[4x pro Jahr]*: 1 (0.4%)
- Following regular parasitological faecal analyses (depending on the results of the **individual faecal samples)** *[nach regelmäßigen parasitologischen Kotuntersuchungen (je nach Ergebnis der Einzelkotproben)]*: 65 (28.5%)
- Following regular parasitological faecal analyses (depending on the results of the **pooled faecal samples)** *[nach regelmäßigen parasitologischen Kotuntersuchungen (je nach Ergebnis der Sammelkotproben)]*: 78 (34.2%)

50. Do you monitor how successful the deworming treatment is? (If "yes" at question 47. Multiple choice. n=228)
[Wird der Entwurmungserfolg kontrolliert? (Wenn „Ja" bei Frage 47. Mehrfachantwort. n=228)]

- No specific control *[nein, keine spezielle Kontrolle]*: 98 (43.0%)
- Yes, by analysing faecal samples after deworming *[ja, mittels parasitologischer Kotprobenuntersuchung nach der Entwurmung]*: 71 (31.1%)
- Yes, by checking the general condition after deworming *[ja, mittels Kontrolle des Allgemeinbefindens nach der Entwurmung]*: 50 (21.9%)
- Yes, by monitoring weight gain after deworming *[ja, mittels Kontrolle der Gewichtszunahme nach der Entwurmung]*: 22 (9.6%)
- No information *[keine Antwort]*: 9 (3.9%)

51. Do you change the drug group for deworming from time to time? (If "yes" at question 47. Single choice. n=228)
[Wechseln Sie die Wirkstoffgruppe zur Entwurmung von Zeit zu Zeit? (Wenn „Ja" bei Frage 47. Einfachantwort. n=228)]

- ➢ Yes *[ja]*: 167 (73.2%)
- ➢ No *[nein]*: 52 (22.8%)
- ➢ No information *[keine Antwort]*: 9 (3.9%)

52. Are additional vitamin and trace element formulations administered as an injection in your stock? (Single choice. n=255)
[Werden in Ihrem Bestand zusätzliche Vitamin- und Spurenelementpräparate per Injektion verabreicht? (Einfachantwort. n=255)]

- ➢ No *[nein]*: 174 (68.2%)
- ➢ Yes *[ja]*: 70 (27.5%)
- ➢ No information *[keine Antwort]*: 11 (4.3%)

53. Which preparations are additionally administered? (If "yes" at question 52. Multiple choice. n=70. Second percentage applies to all participants (n=255))
[Welche Präparate werden zusätzlich verabreicht? (Wenn „Ja" bei Frage 52. Mehrfachantwort. n=70. Zweite Prozentzahl in Bezug auf alle Halter (n=255))]

- ➢ Vitamin E/selenium *[Vitamin E/Selen]*: 57 (81.4% / 22.4%)
- ➢ Vitamin D: 44 (62.9% / 17.3%)
- ➢ Other *[Sonstiges]*: 12 (17.1% / 4.7%)
  - "Vitamin-B-complex" *[Vitamin-B-Komplex]*; "Vitamin ADE" *[Vitamin ADE]*; "Zinc" *[Zink]*; "Vitamin B12" *[Vitamin B12]*; "Phosphor" *[Phosphor]*
- ➢ No information *[keine Angabe]*: 2 (2.9%)

Part E: diseases *[Teil E: Krankheiten]*

54. Please cross whether and how often the listed diseases / disease symptoms occur in your stock (sick animals per year). 1. Digestive tract (Single choice. n=255)
[Bitte kreuzen Sie an, ob und wie häufig gelistete Krankheiten / Krankheitssymptome in Ihrem Bestand auftreten (Anzahl der erkrankten Tiere pro Jahr). 1. Verdauungsapparat (Einfachantwort. n=255)]

- Gastrointestinal parasites *[Magen-Darm-Parasiten]*
  - ➢ Never *[nie]*: 89 (34.9%)
  - ➢ Less than once a year *[seltener als 1x pro Jahr]*: 74 (29.0%)
  - ➢ Once or twice a year *[1-2x pro Jahr]*: 50 (19.6%)
  - ➢ Up to ten times a year *[bis zu 10x pro Jahr]*: 6 (2.4%)
  - ➢ >10 times a year *[>10x pro Jahr]*: 2 (0.8%)
  - ➢ No information *[keine Angabe]*: 34 (13.3%)
- Diarrhoea *[Durchfall]*
  - ➢ Never *[nie]*: 116 (45.5%)
  - ➢ Less than once a year *[seltener als 1x pro Jahr]*: 62 (24.3%)
  - ➢ Once or twice a year *[1-2x pro Jahr]*: 31 (12.2%)
  - ➢ Up to ten times a year *[bis zu 10x pro Jahr]*: 1 (0.4%)
  - ➢ >10 times a year *[>10x pro Jahr]*: 0 (0%)
  - ➢ No information *[keine Angabe]*: 45 (17.6%)
- Colic *[Koliken]*
  - ➢ Never *[nie]*: 161 (63.1%)
  - ➢ Less than once a year *[seltener als 1x pro Jahr]*: 29 (11.4%)

- ➢ Once or twice a year *[1-2x pro Jahr]*: 9 (3.5%)
- ➢ Up to ten times a year *[bis zu 10x pro Jahr]*: 0 (0%)
- ➢ >10 times a year *[>10x pro Jahr]*: 1 (0.4%)
- ➢ No information *[keine Angabe]*: 55 (21.6%)

- Dental issues *[Zahnprobleme]*
  - ➢ Never *[nie]*: 116 (45.5%)
  - ➢ Less than once a year *[seltener als 1x pro Jahr]*: 67 (26.3%)
  - ➢ Once or twice a year *[1-2x pro Jahr]*: 20 (7.8%)
  - ➢ Up to ten times a year *[bis zu 10x pro Jahr]*: 1 (0.4%)
  - ➢ >10 times a year *[>10x pro Jahr]*: 0 (0%)
  - ➢ No information *[keine Angabe]*: 51 (20.0%)

55. Please cross whether and how often the listed diseases / disease symptoms occur in your stock (sick animals per year). 2. Skin (Single choice. n=255)
[Bitte kreuzen Sie an, ob und wie häufig gelistete Krankheiten / Krankheitssymptome in Ihrem Bestand auftreten (Anzahl der erkrankten Tiere pro Jahr). 2. Haut (Einfachantwort. n=255)]

- Ectoparasites (e.g. mange mites) *[Ektoparasiten (z.B. Räudemilben)]*
  - ➢ Never *[nie]*: 87 (34.1%)
  - ➢ Less than once a year *[seltener als 1x pro Jahr]*: 69 (27.1%)
  - ➢ Once or twice a year *[1-2x pro Jahr]*: 60 (23.5%)
  - ➢ Up to ten times a year *[bis zu 10x pro Jahr]*: 6 (2.4%)
  - ➢ >10 times a year *[>10x pro Jahr]*: 3 (1.2%)
  - ➢ No information *[keine Angabe]*: 30 (11.8%)
- Abscesses *[Abszesse]*
  - ➢ Never *[nie]*: 133 (52.2%)
  - ➢ Less than once a year *[seltener als 1x pro Jahr]*: 51 (20.0%)
  - ➢ Once or twice a year *[1-2x pro Jahr]*: 11 (4.3%)
  - ➢ Up to ten times a year *[bis zu 10x pro Jahr]*: 0 (0%)
  - ➢ >10 times a year *[>10x pro Jahr]*: 0 (0%)
  - ➢ No information *[keine Angabe]*: 60 (23.5%)
- Mycosis *[Pilzinfektionen]*
  - ➢ Never *[nie]*: 156 (61.2%)
  - ➢ Less than once a year *[seltener als 1x pro Jahr]*: 31 (12.2%)
  - ➢ Once or twice a year *[1-2x pro Jahr]*: 2 (0.8%)
  - ➢ Up to ten times a year *[bis zu 10x pro Jahr]*: 0 (0%)
  - ➢ >10 times a year *[>10x pro Jahr]*: 0 (0%)
  - ➢ No information *[keine Angabe]*: 66 (25.9%)
- Injuries due to rank fights *[Verletzungen durch Rangordnungskämpfe]*
  - ➢ Never *[nie]*: 126 (49.4%)
  - ➢ Less than once a year *[seltener als 1x pro Jahr]*: 51 (20.0%)
  - ➢ Once or twice a year *[1-2x pro Jahr]*: 23 (9.0%)
  - ➢ Up to ten times a year *[bis zu 10x pro Jahr]*: 2 (0.8%)
  - ➢ >10 times a year *[>10x pro Jahr]*: 0 (0%)
  - ➢ No information *[keine Angabe]*: 53 (20.8%)
- Other skin issues *[Andere Hautprobleme]*
  - ➢ Never *[nie]*: 115 (45.1%)
  - ➢ Less than once a year *[seltener als 1x pro Jahr]*: 47 (18.4%)
  - ➢ Once or twice a year *[1-2x pro Jahr]*: 17 (6.7%)
  - ➢ Up to ten times a year *[bis zu 10x pro Jahr]*: 1 (0.4%)
  - ➢ >10 times a year *[>10x pro Jahr]*: 1 (0.4%)
  - ➢ No information *[keine Angabe]*: 74 (29.0%)

56. Please cross whether and how often the listed diseases / disease symptoms occur in your stock (sick animals per year). 3. Reproduction (Single choice. n=255)
[Bitte kreuzen Sie an, ob und wie häufig gelistete Krankheiten / Krankheitssymptome in Ihrem Bestand auftreten (Anzahl der erkrankten Tiere pro Jahr). 3. Fortpflanzung (Einfachantwort. n=255)]

- Abortions (miscarriages) *[Aborte (Fehlgeburt)]*
  - ➢ Never *[nie]*: 109 (42.7%)
  - ➢ Less than once a year *[seltener als 1x pro Jahr]*: 59 (23.1%)
  - ➢ Once or twice a year *[1-2x pro Jahr]*: 9 (3.5%)
  - ➢ Up to ten times a year *[bis zu 10x pro Jahr]*: 1 (0.4%)
  - ➢ >10 times a year *[>10x pro Jahr]*: 0 (0%)
  - ➢ No information *[keine Angabe]*: 77 (30.2%)
- Stillbirths *[Totgeburten]*
  - ➢ Never *[nie]*: 122 (47.8%)
  - ➢ Less than once a year *[seltener als 1x pro Jahr]*: 48 (18.8%)
  - ➢ Once or twice a year *[1-2x pro Jahr]*: 7 (2.7%)
  - ➢ Up to ten times a year *[bis zu 10x pro Jahr]*: 0 (0%)
  - ➢ >10 times a year *[>10x pro Jahr]*: 0 (0%)
  - ➢ No information *[keine Angabe]*: 78 (30.6%)
- Difficult births *[Schwergeburten]*
  - ➢ Never *[nie]*: 121 (47.5%)
  - ➢ Less than once a year *[seltener als 1x pro Jahr]*: 35 (13.7%)
  - ➢ Once or twice a year *[1-2x pro Jahr]*: 13 (5.1%)
  - ➢ Up to ten times a year *[bis zu 10x pro Jahr]*: 1 (0.4%)
  - ➢ >10 times a year *[>10x pro Jahr]*: 0 (0%)
  - ➢ No information *[keine Angabe]*: 85 (33.3%)
- Stunted growth *[Kümmerer-Fohlen]*
  - ➢ Never *[nie]*: 128 (50.2%)
  - ➢ Less than once a year *[seltener als 1x pro Jahr]*: 35 (13.7%)
  - ➢ Once or twice a year *[1-2x pro Jahr]*: 5 (2.0%)
  - ➢ Up to ten times a year *[bis zu 10x pro Jahr]*: 0 (0%)
  - ➢ >10 times a year *[>10x pro Jahr]*: 0 (0%)
  - ➢ No information *[keine Angabe]*: 87 (34.1%)
- Malformations in crias *[Missbildungen der Fohlen]*
  - ➢ Never *[nie]*: 145 (56.9%)
  - ➢ Less than once a year *[seltener als 1x pro Jahr]*: 20 (7.8%)
  - ➢ Once or twice a year *[1-2x pro Jahr]*: 2 (0.8%)
  - ➢ Up to ten times a year *[bis zu 10x pro Jahr]*: 0 (0%)
  - ➢ >10 times a year *[>10x pro Jahr]*: 0 (0%)
  - ➢ No information *[keine Angabe]*: 88 (34.5%)
- Lack of milk in females *[Milchmangel der Stuten]*
  - ➢ Never *[nie]*: 128 (50.2%)
  - ➢ Less than once a year *[seltener als 1x pro Jahr]*: 31 (12.2%)
  - ➢ Once or twice a year *[1-2x pro Jahr]*: 8 (3.1%)
  - ➢ Up to ten times a year *[bis zu 10x pro Jahr]*: 0 (0%)
  - ➢ >10 times a year *[>10x pro Jahr]*: 0 (0%)
  - ➢ No information *[keine Angabe]*: 88 (34.5%)
- Fertility disorders *[Fruchtbarkeitsstörungen]*
  - ➢ Never *[nie]*: 110 (43.1%)
  - ➢ Less than once a year *[seltener als 1x pro Jahr]*: 41 (16.1%)
  - ➢ Once or twice a year *[1-2x pro Jahr]*: 16 (6.3%)
  - ➢ Up to ten times a year *[bis zu 10x pro Jahr]*: 4 (1.6%)
  - ➢ >10 times a year *[>10x pro Jahr]*: 0 (0%)
  - ➢ No information *[keine Angabe]*: 84 (32.9%)

57. Please cross whether and how often the listed diseases / disease symptoms occur in your stock (sick animals per year). 4. Cardiovascular system (Single choice. n=255)
[Bitte kreuzen Sie an, ob und wie häufig gelistete Krankheiten / Krankheitssymptome in Ihrem Bestand auftreten (Anzahl der erkrankten Tiere pro Jahr). 4. Herz-Kreislauf-Apparat (Einfachantwort. n=255)]

- Pneumonia *[Lungenentzündungen]*
  - ➢ Never *[nie]*: 183 (71.8%)
  - ➢ Less than once a year *[seltener als 1x pro Jahr]*: 33 (12.9%)
  - ➢ Once or twice a year *[1-2x pro Jahr]*: 2 (0.8%)
  - ➢ Up to ten times a year *[bis zu 10x pro Jahr]*: 0 (0%)
  - ➢ >10 times a year *[>10x pro Jahr]*: 0 (0%)
  - ➢ No information *[keine Angabe]*: 37 (14.5%)
- Heart murmurs / heart diseases *[Herzgeräusche / Herzerkrankungen]*
  - ➢ Never *[nie]*: 203 (79.6%)
  - ➢ Less than once a year *[seltener als 1x pro Jahr]*: 9 (3.5%)
  - ➢ Once or twice a year *[1-2x pro Jahr]*: 1 (0.4%)
  - ➢ Up to ten times a year *[bis zu 10x pro Jahr]*: 0 (0%)
  - ➢ >10 times a year *[>10x pro Jahr]*: 0 (0%)
  - ➢ No information *[keine Angabe]*: 42 (16.5%)
- Anaemia *[Anämie (Blutarmut)]*
  - ➢ Never *[nie]*: 180 (70.6%)
  - ➢ Less than once a year *[seltener als 1x pro Jahr]*: 24 (9.4%)
  - ➢ Once or twice a year *[1-2x pro Jahr]*: 9 (3.5%)
  - ➢ Up to ten times a year *[bis zu 10x pro Jahr]*: 1 (0.4%)
  - ➢ >10 times a year *[>10x pro Jahr]*: 0 (0%)
  - ➢ No information *[keine Angabe]*: 41 (16.1%)

58. Please cross whether and how often the listed diseases / disease symptoms occur in your stock (sick animals per year). 5. Musculoskeletal system (Single choice. n=255)
[Bitte kreuzen Sie an, ob und wie häufig gelistete Krankheiten / Krankheitssymptome in Ihrem Bestand auftreten (Anzahl der erkrankten Tiere pro Jahr). 5. Bewegungsapparat (Einfachantwort. n=255)]

- Fractures *[Knochenbrüche]*
  - ➢ Never *[nie]*: 196 (76.9%)
  - ➢ Less than once a year *[seltener als 1x pro Jahr]*: 21 (8.2%)
  - ➢ Once or twice a year *[1-2x pro Jahr]*: 0 (0%)
  - ➢ Up to ten times a year *[bis zu 10x pro Jahr]*: 0 (0%)
  - ➢ >10 times a year *[>10x pro Jahr]*: 0 (0%)
  - ➢ No information *[keine Angabe]*: 38 (14.9%)
- Lameness *[Lahmheiten]*
  - ➢ Never *[nie]*: 147 (57.6%)
  - ➢ Less than once a year *[seltener als 1x pro Jahr]*: 55 (21.6%)
  - ➢ Once or twice a year *[1-2x pro Jahr]*: 15 (5.9%)
  - ➢ Up to ten times a year *[bis zu 10x pro Jahr]*: 0 (0%)
  - ➢ >10 times a year *[>10x pro Jahr]*: 0 (0%)
  - ➢ No information *[keine Angabe]*: 38 (14.9%)
- Exposed injuries *[Offene Verletzungen]*
  - ➢ Never *[nie]*: 147 (57.6%)
  - ➢ Less than once a year *[seltener als 1x pro Jahr]*: 54 (21.2%)
  - ➢ Once or twice a year *[1-2x pro Jahr]*: 15 (5.9%)
  - ➢ Up to ten times a year *[bis zu 10x pro Jahr]*: 1 (0.4%)
  - ➢ >10 times a year *[>10x pro Jahr]*: 0 (0%)
  - ➢ No information *[keine Angabe]*: 38 (14.9%)

- Limb deformities *[Gliedmaßenfehlstellung]*
  - Never *[nie]*: 173 (67.8%)
  - Less than once a year *[seltener als 1x pro Jahr]*: 33 (12.9%)
  - Once or twice a year *[1-2x pro Jahr]*: 2 (0.8%)
  - Up to ten times a year *[bis zu 10x pro Jahr]*: 2 (0.8%)
  - >10 times a year *[>10x pro Jahr]*: 0 (0%)
  - No information *[keine Angabe]*: 45 (17.6%)

59. Please cross whether and how often the listed diseases / disease symptoms occur in your stock (sick animals per year). 6. Other (Single choice. n=255)
[Bitte kreuzen Sie an, ob und wie häufig gelistete Krankheiten / Krankheitssymptome in Ihrem Bestand auftreten (Anzahl der erkrankten Tiere pro Jahr). 6. Sonstiges (Einfachantwort. n=255)]

- Emaciation *[Abmagerung]*
  - Never *[nie]*: 119 (46.7%)
  - Less than once a year *[seltener als 1x pro Jahr]*: 66 (25.9%)
  - Once or twice a year *[1-2x pro Jahr]*: 30 (11.8%)
  - Up to ten times a year *[bis zu 10x pro Jahr]*: 3 (1.2%)
  - >10 times a year *[>10x pro Jahr]*: 0 (0%)
  - No information *[keine Angabe]*: 37 (14.5%)
- Recumbency *[Festliegen]*
  - Never *[nie]*: 176 (69.0%)
  - Less than once a year *[seltener als 1x pro Jahr]*: 25 (9.8%)
  - Once or twice a year *[1-2x pro Jahr]*: 3 (1.2%)
  - Up to ten times a year *[bis zu 10x pro Jahr]*: 0 (0%)
  - >10 times a year *[>10x pro Jahr]*: 1 (0.4%)
  - No information *[keine Angabe]*: 50 (19.6%)
- Central nervous disorders (e.g. walking in circles *[Zentralnervöse Probleme (z.B. im Kreis wandern)]*
  - Never *[nie]*: 196 (76.9%)
  - Less than once a year *[seltener als 1x pro Jahr]*: 9 (3.5%)
  - Once or twice a year *[1-2x pro Jahr]*: 0 (0%)
  - Up to ten times a year *[bis zu 10x pro Jahr]*: 0 (0%)
  - >10 times a year *[>10x pro Jahr]*: 0 (0%)
  - No information *[keine Angabe]*: 50 (19.6%)
- Eye diseases *[Augenkrankheiten]*
  - Never *[nie]*: 127 (49.8%)
  - Less than once a year *[seltener als 1x pro Jahr]*: 48 (18.8%)
  - Once or twice a year *[1-2x pro Jahr]*: 31 (12.2%)
  - Up to ten times a year *[bis zu 10x pro Jahr]*: 1 (0.4%)
  - >10 times a year *[>10x pro Jahr]*: 1 (0.4%)
  - No information *[keine Angabe]*: 47 (18.4%)

60. Do other diseases/symptoms of disease occur frequently in your stock that are not listed here and that seem important to you? Please specify them here. (Free text, each named once)
[Kommen in Ihrem Bestand andere Krankheiten/Krankheitssymptome häufig vor, die hier nicht gelistet wurden und die Ihnen wichtig erscheinen? Bitte geben Sie diese hier an. (Freitext, je einmal genannt)]

- "Adipositas" *[Adipositas]*; "Arthrosis" *[Arthrose]*; "Bluetongue virus infection" *[Blauzungenvirus-Infektion]*; "Borna disease virus infection" *[Bornavirus-Infektion]*; "Calcinosis" *[Kalzinose]*; "Chronic obstructive pulmonary disease" *[chronisch obstruktive Lungenerkrankung]*; "Cystitis" *[Cystitis]*; "Excess testosterone" *[Testosteronüberschuss]*; "Intestinal obstruction" *[Darmverschluss]*; "Intoxication" *[Vergiftung]*; "Mycoplasma infection" *[Mykoplasmeninfektion]*; "Oesophageal obstruction" *[Schlundverstopfung]*; "Otitis" *[Otitis]*; "Shearing injury" *[Schurverletzung]*; "Tumours" *[Tumore]*;

"Urolithiasis" *[Urolithiasis]*; "Uterus torsion" *[Uterustorsion]*; "Zinc/selenium deficiency" *[Zink-/Selenmangel]*

61. Are you registered with and looked after by a veterinary practice? (Single choice. n=255)
[Werden Sie von einer festen Tierarztpraxis betreut? (Einfachantwort. n=255)]

- ➢ Yes *[ja]*: 223 (87.5%)
- ➢ No *[nein]*: 12 (4.7%)
- ➢ Different veterinary practices *[wechselnde Tierarztpraxen]*: 15 (5.9%)
- ➢ No information *[keine Antwort]*: 5 (2.0%)

62. What kind of veterinary practice is it at present? (If "yes", "different veterinary practices" at question 61. Single choice. n=238)
[Um was für eine Tierarztpraxis handelt es sich derzeitig? (Wenn „Ja", „Wechselnde Tierarztpraxen" bei Frage 61. Einfachantwort. n=238)]

- ➢ Mixed practice (Large and small animal practice) *[Gemischtpraxis (Klein- und Großtiere)]*: 140 (58.8%)
- ➢ Farm animal practice *[Praxis für Nutztiere]*: 44 (18.5%)
- ➢ Specialised camelid practice *[spezialisierte Praxis für Neuweltkameliden]*: 23 (9.7%)
- ➢ Equine practice *[Praxis für Pferde]*: 15 (6.3%)
- ➢ Small animal practice *[Praxis für Kleintiere]*: 4 (1.7%)
- ➢ Other *[Sonstiges]*: 11 (4.6%)
    - "Practice not specialised in South American camelids but with acquired knowledge" *[Praxen, die sich über Neuweltkamele fortbilden, aber ohne direkte Spezialisierung]*; "Veterinarian from other European country" *[Tierarzt aus dem europäischen Ausland]*
- ➢ No information *[keine Antwort]*: 1 (0.4%)

63. How often does your current veterinarian visit on average? (If "yes", "different veterinary practices" at question 61. Single choice. n=238)
[Wie häufig kommt Ihr derzeitiger Haustierarzt / Ihre derzeitige Haustierärztin im Schnitt? (Wenn „Ja", „Wechselnde Tierarztpraxen" bei Frage 61. Einfachantwort. n=238)]

- ➢ Once a year *[1x pro Jahr]*: 54 (22.7%)
- ➢ Twice a year *[2x pro Jahr]*: 71 (29.8%)
- ➢ Three times a year *[3x pro Jahr]*: 41 (17.2%)
- ➢ Four times a year *[4x pro Jahr]*: 48 (20.2%)
- ➢ Monthly *[monatlich]*: 18 (7.6%)
- ➢ Several times a month *[mehrmals im Monat]*: 2 (0.8%)
- ➢ No information *[keine Antwort]*: 4 (1.7%)

64. Do you feel well cared for by your vet and do you think that this practice possesses sufficient knowledge about camelids? (If "yes", "different veterinary practices" at question 61. Single choice. n=238)
[Fühlen Sie sich von Ihrem Haustierarzt / Ihrer Haustierärztin gut betreut und haben Sie das Gefühl, dass diese Praxis ausreichend Wissen über Neuweltkameliden hat? (Wenn „Ja", „Wechselnde Tierarztpraxen" bei Frage 61. Einfachantwort. n=238)]

- ➢ Yes *[ja]*: 172 (72.3%)
- ➢ No *[nein]*: 58 (24.4%)
- ➢ No information *[keine Antwort]*: 8 (3.4%)

65. Please rate the following statement: "Veterinarians in general possess sufficient knowledge to be able to treat South American camelids." (Single choice. n=255)
[Bitte bewerten Sie folgende Aussage: „Tierärztinnen und Tierärzte im Allgemeinen haben ausreichend Wissen zur Behandlung von Neuweltkameliden" (Einfachantwort. n=255)]

- ➢ Strongly agree *[stimme voll zu]*: 10 (3.9%)
- ➢ More likely to agree *[stimme eher zu]*: 18 (7.1%)
- ➢ In part *[teils teils]*: 80 (31.4%)
- ➢ Agree less *[stimme weniger zu]*: 84 (32.9%)
- ➢ Disagree *[stimme gar nicht zu]*: 56 (22.0%)
- ➢ No information *[keine Antwort]*: 7 (2.7%)

66. How would you rate your own knowledge of the main health problems of South American camelids?
Single choice. n=255)
[Wie schätzen Sie Ihre eigenen Kenntnisse über die wichtigsten Gesundheitsprobleme bei Neuweltkameliden ein? (Einfachantwort. n=255)]

- ➢ Outstanding knowledge *[sehr gut]*: 37 (14.5%)
- ➢ Good knowledge *[gut]*: 141 (55.3%)
- ➢ Moderate knowledge *[mäßig]*: 70 (27.5%)
- ➢ Poor knowledge *[schlecht]*: 2 (0.8%)
- ➢ No information *[keine Antwort]*: 5 (2.0%)

67. Please evaluate to what extent veterinarians provide information about health problems of camelids.
(Single choice. n=255)
[Bitte bewerten Sie das Vorhandensein von Informationsmaterial über Gesundheitsprobleme bei Neuweltkameliden seitens der Tierärztinnen und Tierärzte. (Einfachantwort. n=255)]

- ➢ Absolutely sufficient *[vollkommen ausreichend]*: 7 (2.7%)
- ➢ Sufficient *[ausreichend]*: 29 (11.4%)
- ➢ Mediocre *[teils teils]*: 68 (26.7%)
- ➢ Insufficient *[zu wenig]*: 99 (38.8%)
- ➢ Far too insufficient *[viel zu wenig]*: 46 (18.0%)
- ➢ No information *[keine Antwort]*: 6 (2.4%)

68. Please evaluate to what extent veterinary offices provide information about health problems of camelids.
(Single choice. n=255)
[Bitte bewerten Sie das Vorhandensein von Informationsmaterial über Gesundheitsprobleme bei Neuweltkameliden seitens der Veterinärämter. (Einfachantwort. n=255)]

- ➢ Absolutely sufficient *[vollkommen ausreichend]*: 1 (0.4%)
- ➢ Sufficient *[ausreichend]*: 16 (6.3%)
- ➢ Mediocre *[teils teils]*: 54 (21.2%)
- ➢ Insufficient *[zu wenig]*: 91 (35.7%)
- ➢ Far too insufficient *[viel zu wenig]*: 79 (31.0%)
- ➢ No information *[keine Antwort]*: 14 (5.5%)
